# Supplementary material for: The Impact of Rural Hospital Closures and Health Service Restructuring on Provincial- and Community-Level Patterns of Hospital Admissions in New Brunswick
Source: Int J Environ Res Public Health. 2022 Jun 14;19(12):7258. doi: 10.3390/ijerph19127258 (PMC9223870; doi:10.3390/ijerph19127258)
Supplement: Supplementary file 1 [file ijerph-19-07258-s001.zip › ijerph-1746136-supplementary.pdf]

Table S1: coding for Ambulatory Care Sensitive Conditions inclusion and exclusion criteria.

**Inclusion**	
**Health Condition**	**ICD 10 Codes**
Grand mal status and other epileptic convulsions	G40, G41
Chronic obstructive pulmonary disease (COPD)	J41, J42, J43, J44, or J47
Asthma	J45
Congestive heart failure and pulmonary edema (CHF)	I50, J81
Hypertension	I10.0, I10.1, I11
Angina	I20, I23.82, I24.0, I24.8, I24.9
Diabetes	E10.0, E10.1, E10.63, E10.9, E11.0, E11.1, E11.63, E11.9, E13.0, E13.1, E13.63, E13.9, E14.0, E14.1, E14.63, E14.9, E10.64, E11.64, E13.64, E14.64
**Exclusion**	
Any hospitalization record with the following Canadian Classification of Health Interventions (CCI) codes:<br><br>1HA58, 1HA80, 1HA87, 1HB53, 1HB54, 1HB55, 1HB87, 1HD53, 1HD54, 1HD55, 1HH59, 1HH71, 1HJ76, 1HJ82, 1HM57, 1HM78, 1HM80, 1HN71, 1HN80, 1HN87, 1HP76, 1HP78, 1HP80, 1HP82, 1HP83, 1HP87, 1HR71, 1HR80, 1HR84, 1HR87, 1HS80, 1HS90, 1HT80, 1HT89, 1HT90, 1HU80, 1HU90, 1HV80, 1HV90, 1HW78, 1HW79, 1HX71, 1HX78, 1HX79, 1HX80, 1HX83, 1HX86, 1HX87, 1HY85, 1HZ53 rubric (except 1HZ53LAKP), 1HZ54, 1HZ55 rubric (except 1HZ55LAKP), 1.HZ.56, 1.HZ.57, 1HZ59, 1HZ80, 1HZ85, 1HZ87, 1IF83, 1IJ50, 1IJ54GQAZ, 1IJ55, 1IJ57, 1IJ76, 1IJ80, 1IK57, 1IK80, 1IK87, 1IN84, 1LA84, 1LC84, 1LD84, 1YY54LANJ	
